# Role of Prucalopride in Treating Functional Constipation and Gastroparesis: A Systemic Review

**DOI:** 10.7759/cureus.14306

**Published:** 2021-04-05

**Authors:** Hassam Ali, Rahul Pamarthy, Shiza Sarfraz

**Affiliations:** 1 Internal Medicine, East Carolina University, Vidant Medical Center, Greenville, USA; 2 Anesthesiology, Bahawal Victoria Hospital, Quaid-E-Azam Medical College, Bahawalpur, PAK

**Keywords:** prucalopride, functional constipation, gastroparesis, chronic constipation, diabetic gastroparesis, serotonin agonists, resolor

## Abstract

Prucalopride is a selective serotonin receptor agonist that can be used to treat chronic constipation. This article reviews the clinical efficacy side effects of prucalopride, assessing its role in constipation and gastroparesis. Relevant published medical literature was identified by using the search terms "constipation," "gastroparesis," and "prucalopride" from 2010 and onwards. The databases included PubMed/MEDLINE and EMBASE. Bibliographies from published literature and websites were also reviewed. Results were filtered for English language and randomized controlled trials. Out of the 18 results, abstracts were manually reviewed for studies with similar statistical methodology; eight studies were selected for constipation and two studies for gastroparesis.

In two four-week trials, prucalopride showed improvement in gastric emptying and the gastroparesis cardinal symptom index over placebo, with a 1-4 mg/day dosage. In seven 12-week trials in patients with chronic constipation, oral prucalopride 2-4 mg/day was more significant than placebo to improve the number of bowel movements and symptoms. One study showed no significant bowel function differences when prucalopride was compared to placebo over 12 or 24 weeks.

Prucalopride was generally well-tolerated, and the most common adverse events reported were headache, nausea, diarrhea, and abdominal pain. Further long-term and comparative data would be beneficial to show that prucalopride can be an advantageous treatment option for patients with chronic idiopathic constipation (CIC) or gastroparesis. Additionally, it would be interesting to see its effect on irritable bowel syndrome-constipation predominant, as it has some overlap with idiopathic constipation.

## Introduction and background

One of the most common gastrointestinal (GI) complaints among the general population is constipation, resulting in higher inpatient and outpatient costs and overtreatment [1]. Less often, constipation is secondary to reversible causes like diabetes, electrolyte disturbance, hypothyroidism, obstruction, or medications. Constipation without any apparent underlying etiology can be classified as idiopathic or functional constipation. It often involves difficult and incomplete defecation. Idiopathic constipation should not come under the irritable bowel syndrome with constipation (IBS-C) criteria [2]. Rome IV criteria [3] defines chronic idiopathic constipation (CIC) as including two or more of the following symptoms for the last three months, and symptom onset should have been at least six months prior to diagnosis: 1) Straining during >25% of defecations; 2) Lumpy or hard stools in >25% of defecations; 3) Sensation of incomplete evacuation in >25% of defecations; 4) Sensation of anorectal obstruction/blockage in >25% of defecations; 5) Manual maneuvers (e.g., digital evacuation, support of the pelvic floor) in >25% of evacuations; and 6) Fewer than three spontaneous bowel movements per week.

In the western world, constipation has shown to be more predominant in females (34% as compared to 26% in men), older adults over the age of 65, inadequate physical activity, and low socioeconomic status [3-6]. Treatment for chronic constipation of idiopathic etiology initially involves dietary and lifestyle changes; medications commonly utilized could be osmotic laxatives or gut stimulants [7]. In the recent decade or so, prucalopride, a selective, high-affinity serotonin (5-HT4) receptor agonist with prokinetic properties has been shown to improve the symptoms of chronic constipation [7]. Prucalopride has also been shown to accelerate gastric emptying and improve the frequency of bowel movement frequency [8].

Mechanism of action of serotonin receptors in chronic constipation and gastroparesis

The serotonin receptors are present in abundance in the GI tract, located in the myenteric plexus, enterochromaffin cells, and smooth muscle cells. 5-HT4 receptors are G-protein coupled, which increases cyclic adenosine monophosphate (cAMP) production when stimulated by serotonin agonists like prucalopride, resulting in neurotransmitters modulation [9]. One of these neurotransmitters is acetylcholine, whose excitatory effects on the GI tract is thought to be the primary mechanism of 5-HT4 receptor agonists [9-10]. Acetylcholine results in contraction of the longitudinal muscle layer and circular muscle layer relaxation leading to the advancement of luminal contents [10-11]. Prucalopride has a high affinity for the 5-HT4 receptor only, without interacting with other erotogenic receptors at therapeutic doses. Regular bowel movement is characterized by migratory high amplitude contractions traversing the colon's length, resulting in defecation [12]. The absence, reduction in frequency, or decrease in these contractions' amplitude can lead to constipation [13]. Healthy human subjects have shown to exhibit an increase in overall colonic transit and acceleration while on prucalopride; on the other hand, in people with chronic constipation, studies show that it improves gastric motility and small bowel transit [14-15]. 

Some of the initial 5-HT4 receptor agonists like tegaserod and cisapride showed positive inotropic effects on human isolated myocardial trabeculae. However, prucalopride does not result in changes in late repolarizations, refractory periods, or inciting arrhythmias at therapeutic doses [16]. At supratherapeutic doses, prucalopride can act as a partial agonist of L-type calcium channels and has been shown to interact with the human Ether-à-go-go-Related Gene (hERG), which encodes potassium channels that are essential for regular electrical activity in the heart [16].

## Review

Materials and methods

This review was initiated and summarized per the Preferred Reporting Items for Systematic Review and Meta-Analyses (PRISMA) guidelines.

Eligibility Criteria

The clinical trials that studied the effect of prucalopride on constipation were to have a similar study protocol and included patients of age greater than 18, including males and females, with chronic idiopathic constipation. Constipation was defined as two or fewer spontaneous complete bowel movements (SCBMs) per week. This must be for at least six months before the screening visit. The listed patients were to have either hard stools, rectal tenesmus, or defecation that required straining in at least 25% of bowel movements. Patients with constipation due to secondary causes, such as medication, electrolyte disturbances, neurologic or metabolic diseases, or surgical history, were not included. The primary endpoint or treatment response was defined as an average of ≥3 SCBMs/week. The secondary endpoint was the portion of patients with an increase of spontaneous complete bowel movement above their baseline. The clinical trials that studied the effect of prucalopride on gastroparesis were to have a similar study protocol and included patients of age greater than 18, including males and females, with an established diagnosis of gastroparesis. Patients with gastroparesis due to secondary causes, such as medication or surgical history, were excluded. Primary outcome measures were the gastroparesis cardinal symptom index (GCSI) at the end of treatment, assessing symptom severity change.

Search Strategies and Information Sources

Relevant published medical literature was identified by using the following search terms: "constipation," "gastroparesis," "prucalopride," "Resolor" from January 2010 to January 2021. The databases included PubMed/MEDLINE, EMBASE, and Cochrane. Additionally, bibliographies from published literature and websites were also reviewed.

Study Selection

**Figure 1:**
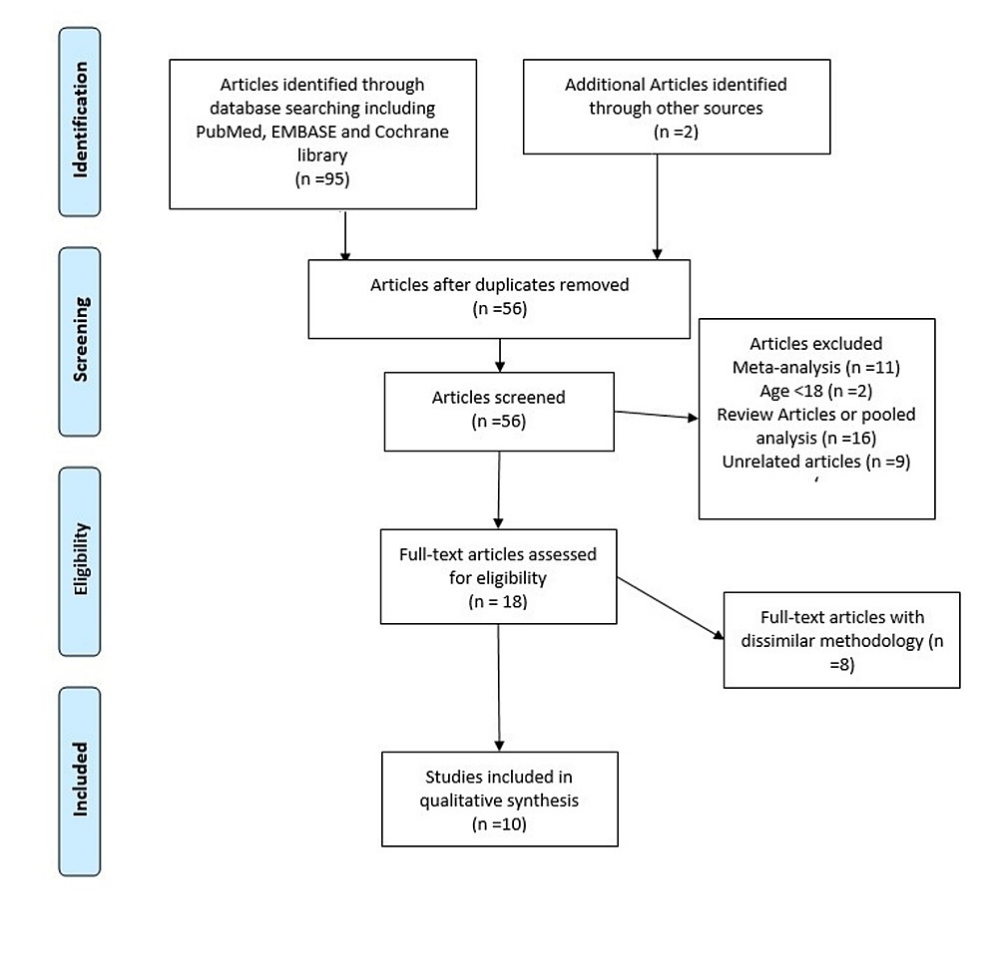
Flowsheet for data selection

From the initial 97 results, 56 were selected after removing duplicate results. Thirty-eight (38) articles were excluded (Figure 1), and 18 full-text articles were manually reviewed for studies with similar statistical methodology; eight studies were selected (Table 1), which compared prucalopride and placebo for constipation, and two studies were included for prucalopride and gastroparesis/colonic motility (Table 2).

**Table 1:** Trials which studied the effect of prucalopride in chronic idiopathic constipation SCBM: spontaneous complete bowel movements, PAC-SYM: patient assessment of constipation symptoms

Article	Duration	Study type	Number of Patients	Main Results	Common adverse effects
Müller-Lissner S et al. [17]	4 weeks	International multicenter, parallel‐group, placebo‐controlled study	300	48.7% (4 mg prucalopride) vs. 26.1% placebo had ≥3 SCBM/week but only during the first week (P≤0.05).	Headache and gastrointestinal symptoms
Ke M et al. [18]	12 weeks	Randomized, placebo-controlled, parallel-group study	501	33.3% in the prucalopride group vs 10.3% for placebo had a weekly average of ≥3 SCBMs during the 12-week treatment (P<0.001).	Diarrhea, nausea, abdominal pain, and headache
Yiannakou Y et al. [19]	12 weeks	Randomized, Double-Blind, Placebo-Controlled study	374	≥3 SCBM/week in 37.9% of prucalopride group (1-2 mg) vs 17.7% for placebo (P<0.0001).	Diarrhea, nausea, abdominal pain, and headache
Quigley EM et al. [20]	12 weeks	Randomized, double-blind, placebo-controlled study	641	23.9% (2 mg) and 23.5% (4 mg) patients reported ≥3 SCBM/week, respectively, as compared to the placebo (12.1%) at week 12 (P≤0.01).	Headache, abdominal pain, nausea, and diarrhea
Camilleri M et al. [21]	12 weeks	Multicenter, randomized, placebo-controlled, parallel-group study	620	30.9% (2 mg) and 28.4% (4 mg) vs. 12.0% in placebo group reported ≥3 SCBM/week (P<0.001).	Headache and abdominal pain
Tack J et al. [22]	12 weeks	Multicenter, randomized, placebo-controlled, parallel-group study	713	9.5% (2 mg prucalopride) (p<0.01), 23.6% for (4 mg prucalopride) (p<0.001) vs. 9.6% for placebo had ≥3 SCBM/week.	Headache, nausea, abdominal pain, and diarrhea
Tack, J. et al. [23]	12 weeks	Double‐blind randomized controlled study	936 women	34.9% (2 mg prucalopride vs. 20.8% (placebo) showed symptom improvement per baseline PAC‐SYM score (P<0.001).	Nausea, diarrhea, abdominal pain, and headache
Piessevaux H et al. [24]	24 weeks	Randomized, parallel‐group, double‐blind, placebo‐controlled study	361	Over the 24‐week period, the result was not statistically different (p = 0.367) between the prucalopride (25.1%) and placebo (20.7%) treatment groups. No statistical significance for the 13–24-week period (prucalopride, 28.1%; placebo, 23.7%; P=0.275).	Abdominal pain and diarrhea

**Table 2:** Trials which studied the effect of prucalopride in gastroparesis GCSI: gastroparesis cardinal symptom index

Article	Duration	Study type	Number of patients	Main Results	Common adverse effects
Andrews CN et al. [8]	4 weeks	Double‐blind crossover trial	15 patients	Rapid gastric emptying (21.9 ± 6.2%) in the prucalopride period vs. placebo (40.0 ± 9.2%) (P = 0.05). Weekly (unadjusted) mean bowel movement (BM) frequency significantly higher in prucalopride (mean 10.5 ± 1.8 BM wk−1) vs placebo (mean 7.5 ± 0.8 BM wk−1), (P < 0.0001)	Headache, abdominal cramping, and diarrhea
Carbone F et al. [25]	4 weeks	Double-blind, randomized, placebo-controlled crossover study	34 patients	Entire gastroparesis study population: Total GCSI (1.65 ± 0.19 vs 2.28 ± 0.20, P < 0.0001). Idiopathic gastroparesis subgroup: Total GCSI (1.81 6 0.21 vs 2.47 6 0.19, P < 0.001)	Nausea and headache

Results

Prucalopride for the Treatment of Chronic Idiopathic Constipation

Müller-Lissner S et al. studied how prucalopride can affect constipation in the elderly (age>65) within four weeks instead of 12 while comparing 1-4 mg doses of prucalopride to placebo [17]. During the first week, 48.7% of patients on 4 mg prucalopride achieved > or =3 SCBM as compared to placebo (26.1%) (P<0.05), however, the results were not statistically significant for four weeks when the percentages were 31% vs. 25%. Although prucalopride was showed signiﬁcant improvement in bowel movement at one week, sadly, this result was not observed at lower doses of prucalopride or at 1-2 weeks. Total PAC‐SYM stool symptoms score was significantly higher in patients treated with 1 or 4 mg prucalopride vs. (P ≤ 0.05). Individual scores and subscores were not mentioned. The proportion of patients with an average increase of ≥1 SCBM/week above baseline, prucalopride showed beneﬁts over placebo at 1 mg, 2 mg, and 4 mg doses after the ﬁrst week. It is essential to know that this was the criterion on which tegaserod was approved for constipation by the FDA in 2002, which was withdrawn later from the market due to cardiovascular risk concerns.

Ke M et al. showed that prucalopride is significantly effective in helping a ≥3 SCBMs therapeutic gain of 23.0% (95% Confidence Interval (CI) = 16.1-30.0%; P < 0.001) over placebo in 12 weeks [18]. Over the first four weeks, an average of ≥3 SCBMs was attained by 34.5% of patients vs. 11.1% on placebo, representing a therapeutic gain of 23.4% (95% CI = 16.4-30.5%; P < 0.001) with prucalopride. The overall mean patient assessment of overall constipation-symptom score (PAC-SYM) was 1.5 at baseline for patients in the placebo and prucalopride groups. The mean change from baseline (lower is better) in the PAC-SYM overall score at 12 weeks was greater in the prucalopride group (0.8 (−0.7)) than in the placebo group (1.2 (−0.4)) at the final on-treatment assessment; (P <0.001). Additionally, the PAC-SYM stool symptoms score also showed statistical significance at 12 weeks. The mean baseline for placebo was 2.2 vs. 2.1 for prucalopride at the start of treatment. Overall, the PAC-SYM stool symptoms score was improved in prucalopride 1.2 (−1.0) vs. placebo 1.7 (-0.5) (P<0.001). Abdominal and rectal symptoms also showed statistical significance in the prucalopride group vs. placebo [18].

The study by Yiannakou Y et al. demonstrated ≥3 SCBMs in 37.9% of patients on prucalopride compared to 17.7% in the placebo group (P<0.0001) [19]. The mean PAC-SYM overall score was 1.75 (SD 0.67) at baseline for the placebo group and 1.84 (SD 0.66) in the prucalopride group. The mean improvement from baseline was greater in the prucalopride group (-0.76; SD 0.77) than in the placebo group (-0.59; SD 0.76) at the final assessment; however, this was not statistically significant (P=0.0623). The PAC-SYM rectal and abdominal symptoms also failed to show any statistical significance between the treatment and placebo group [19].

Quigly EM et al. demonstrated in their study that for 2 mg and 4 mg prucalopride, 23.9% (2 mg) and 23.5%(4 mg) of patients reported ≥3 SCBM/week, respectively, as compared to placebo (12.1%) at week 12 (P≤0.01) [20]. Forty-two point six percent (42.6%) (2 mg) and 46.6% (4 mg) achieved ≥1 SCBM/week at week 12 as compared to placebo (27.5%) (P≤0.001). The proportions of patients with an improvement from baseline of ≥1 point in the overall PAC‐SYM score was higher in the prucalopride vs. placebo group at week 4 (P ≤0.001) in the 2 mg and 4 mg groups and week 12 in the 2 mg group only (P ≤0.001). Overall PAC‐SYM symptoms score for the placebo, 2 mg, and 4 mg groups at the beginning of the trial was 1.97, 2.04, and 1.84, respectively. At 12 weeks, the mean with mean change was 1.52 (−0.45), 1.26 (−0.78) (P<0.001), and 1.28 (−0.56) (P<0.01), respectively. The PAC‐SYM abdominal symptoms score showed a statistically significant improvement in the 2 mg and 4 mg groups at 12 weeks vs. placebo. However, no statistically significant improvement was seen in the rectal symptoms score at the 4 mg dose at the end of 12 weeks. The 2 mg group did show significant improvement over placebo for the rectal symptom subscore (P≤0.05) [20].

In the study by Camilleri M et al. [21], 30.9% receiving 2 mg of prucalopride, 28.4% receiving 4 mg, and 12.0% receiving placebo (P <0.001) reported ≥3 SCBM/week (P <0.001). Additionally, 47.3% (2 mg) of and 46.6% (4 mg) had ≥1 SCBM/week as compared with 25.8% in the placebo group (P <0.001). At baseline, the mean PAC-SYM score for the placebo, 2 mg, and 4 mg groups was 2.0, 1.9, and 1.9, respectively. By the end of 12 weeks, the mean with the change from baseline was 1.6 (-0.4), 1.3 (-0.6), and 1.2 (-0.7), respectively (P<0.001). The abdominal symptom subscore showed significant improvement from baseline at 2 mg and 4 mg vs. prucalopride by 12 weeks (P<0.001). However, no statistically significant improvement was seen in the rectal symptom subscore, although the 2 mg group showed an upward trend with a mean baseline of 1.2 at the start (placebo 1.0) and a mean of 0.6 at 12 weeks (mean change -0.5) (P<0.05).

Averaged over 12 weeks, Tack J et al. demonstrated that a larger number of patients in the prucalopride 2 mg group (19.5%; P<0.01) and 4 mg group (23.6%; P<0.001) had ≥3 SCBM/week as compared with placebo (9.6%) [22]. The overall PAC-SYM symptoms score for the placebo, 2 mg, and 4 mg groups at the start of the trial was 2.06, 2.12, and 2.00. After 12 weeks, the mean with the change from baseline was 1.69 (−0.37), 1.44 (−0.66), and 1.29 (−0.71) (P <0.001). The abdominal and rectal symptom subscore was also significantly improved in the 2 mg (P <0.01) and 4 mg (0.001) groups as compared to placebo. In another trial, Tack J et al. studied the effect of prucalopride on the PAC-SYM severity score in women with chronic constipation [23]. The overall mean PAC‐SYM score at baseline was 2.07 in the placebo group vs. 2.10 in the 2 mg prucalopride group. At week 12, the mean with a mean change from baseline for placebo was 1.70 (−0.36) vs. 1.40 (−0.70) (P <0.001) in the prucalopride group. The abdominal, stool, and rectal subscores showed statistically significant improvement at 12 weeks on 2 mg of prucalopride vs. placebo (P<0.001). At 12 weeks, 34.9% in the prucalopride 2 mg group vs. 20.8% in the placebo group (P<0.001) showed symptom improvement per baseline PAC‐SYM.

Piessevaux H et al. was the first to study the effects of prucalopride (2 mg) over 24-week periods than the previous 12-week studies [24]. Surprisingly, this trial did not show any statistically significant benefit of prucalopride over placebo over 24 weeks or 12 weeks and achieved ≥3 SCBM/week (P = 0.367). No statistical significance was seen in the overall PAC-SYM score for the placebo vs. prucalopride group. In the beginning, the baseline score was 1.97 (placebo) vs. 1.27 (prucalopride). At the end of 24 weeks, the mean and change from mean were 1.29 (−0.68) for placebo vs. 1.27 (−0.55) in the prucalopride group (0.035). Similarly, no significant improvement was seen in rectal (P=0.219) or abdominal subscores (P=0.185).

Prucalopride for Treatment of Gastroparesis

Andrews CN et al. employed patients who were experiencing diabetic gastroparesis or connective tissue disease exclusively [8]. The baseline GCSI for placebo was 2.81 ± 0.34 vs. the 4 mg prucalopride group's 3.29 ± 0.26. After four weeks, GCSI showed improvement in the prucalopride (2.73 ± 0.19) and placebo (2.49 ± 0.27) treatment groups but without statistical significance (P>0.2). However, gastric emptying was more rapid in the prucalopride group (21.9 ± 6.2%) vs. placebo (40.0 ± 9.2%) (P=0.05). Interestingly, weekly mean bowel movement (BM) frequency was significantly higher in prucalopride (mean 10.5 ± 1.8 BM/week) vs. placebo (mean 7.5 ± 0.8 BM/week) (P<0.000) [8].

Carbone F et al. also studied the effect of prucalopride over four weeks for gastroparesis [25]. The results for the entire gastroparesis group and idiopathic gastroparesis were reported separately. The GCSI was significantly improved in the treatment vs. placebo group (prucalopride (1.65 ± 0.19) vs (placebo (2.28 ± 0.2) (P < 0.0001). All GCSI subscales, including nausea/vomiting, satiety, and bloating, were also significantly improved than in placebo. Prucalopride showed improved solid food gastric emptying as compared to placebo (126 ± 13 minutes, P = 0.02). The patient assessment of upper gastrointestinal symptom severity (PAGI-SYM) subscales also showed significant improvement over placebo. The total symptoms for prucalopride vs. placebo were (76.3 [7.75; 110.25] and 47.9 [1.75; 61.25] (P=0.02) for postprandial fullness (17.65 [0.75; 25.0] vs 9.7 [0.0; 9.25] (P=0.03), and for bloating (22.0 [1.5; 36.75] vs 12.6 [0.0; 17.75] (P=0.03). For the idiopathic gastroparesis group, total GCSI showed significant improvement in the prucalopride group when compared with placebo treatment (1.81 ± 0.21 vs. 2.47 ± 0.19, P < 0.001). The PAGI-SYM subscores between prucalopride and placebo were reported as: fullness/satiety (2.37 ± 0.29 vs 3.14 ± 0.25, P<0.0005), bloating/distension (1.82 ± 0.31 vs 2.66 ± 0.30, P<0.0005), nausea/vomiting (1.07 ± 0.22 vs 1.45 ± 0.25, P=0.02), and reflux (1.38 ± 0.25 vs 1.67 ± 0.22, P=0.02). In both the total and idiopathic gastroparesis group, the change in GCSI or PAGI-SYM scores and the gastric emptying rate were not statistically significant (P = >0.05) [25]. 

Adverse Effects of Prucalopride Usage

Our literature review revealed that prucalopride was generally well-tolerated in patients with chronic constipation. The most common adverse events in prucalopride (≤2 mg/day) include nausea, headache, abdominal distention/pain, and diarrhea [26]. These appeared to be transient, primarily at the start of treatment, and of mild severity. Thirty-nine percent (39%)-81 % of patients on prucalopride (≤2 mg) vs. 34%-71% on placebo experienced adverse events [18-23]. An integrated analysis of data from four trials revealed that headache, diarrhea, and nausea were significantly more with prucalopride vs. placebo [18-21]. Surprisingly, abdominal distention was not in them (P<0.001) [26]. In the same analysis, women had a greater risk of nausea than men (P<0.05), and headache was more predominant in younger patients (P<0.001). The study by Cinca et al. noted the incidence of at least one adverse event was 85% (prucalopride) versus 68% (PEG-3350 + electrolytes), respectively [26]; and at least one serious adverse event occurred in 1% of the prucalopride group vs. 0% of PEG-3350 + electrolytes.

## Conclusions

Prucalopride treatment should be recommended in patients with CIC who have not experienced symptom improvement following lifestyle and dietary changes and the use of any previous over-the-counter prescriptions or laxatives. Further long-term and comparable data, including a meta-analysis of current prucalopride trials for gastroparesis, will also be helpful. It would be helpful for prospective future trials to study the effects of prucalopride in patients with concurrent gastroparesis and CIC, although this might demand an increase in dosage. No data are currently available regarding the use of prucalopride for IBS-C or its effect or role in controlling pain, which plays a factor in IBS-C despite its potential overlap with chronic idiopathic constipation.
